# Identification of acute myocardial infarction and stroke events using the National Health Insurance Service database in Korea

**DOI:** 10.4178/epih.e2024001

**Published:** 2023-12-26

**Authors:** Minsung Cho, Hyeok-Hee Lee, Jang-Hyun Baek, Kyu Sun Yum, Min Kim, Jang-Whan Bae, Seung-Jun Lee, Byeong-Keuk Kim, Young Ah Kim, JiHyun Yang, Dong Wook Kim, Young Dae Kim, Haeyong Pak, Kyung Won Kim, Sohee Park, Seng Chan You, Hokyou Lee, Hyeon Chang Kim

**Affiliations:** 1 Department of Public Health, Yonsei University Graduate School, Seoul, Korea; 2 Department of Preventive Medicine, Yonsei University College of Medicine, Seoul, Korea; 3 Department of Internal Medicine, Yonsei University College of Medicine, Seoul, Korea; 4 Institute for Innovation in Digital Healthcare, Yonsei University, Seoul, Korea; 5 Department of Neurology, Kangbuk Samsung Hospital, Sungkyunkwan University School of Medicine, Seoul, Korea; 6 Department of Neurology, Chungbuk National University Hospital, Cheongju, Korea; 7 Department of Internal Medicine, Chungbuk National University College of Medicine, Cheongju, Korea; 8 Division of Cardiology, Department of Internal Medicine, Chungbuk National University Hospital, Cheongju, Korea; 9 Division of Cardiology, Severance Cardiovascular Hospital, Yonsei University College of Medicine, Seoul, Korea; 10 Division of Digital Health, Yonsei University Health System, Seoul, Korea; 11 Department of Medical Records, Severance Hospital, Yonsei University Health System, Seoul, Korea; 12 Department of Information and Statistics, Research Institute of Natural Science, Gyeongsang National University, Jinju, Korea; 13 Department of Neurology, Yonsei University College of Medicine, Seoul, Korea; 14 Institute of Health Insurance & Clinical Research, National Health Insurance Service Ilsan Hospital, Goyang, Korea; 15 Department of Pediatrics, Yonsei University College of Medicine, Seoul, Korea; 16 Department of Health Information & Biostatistics, Graduate School of Public Health, Yonsei University, Seoul, Korea; 17 Department of Biomedical Systems Informatics, Yonsei University College of Medicine, Seoul, Korea

**Keywords:** Acute myocardial infarction, Stroke, Identification, Algorithm, Epidemiology

## Abstract

**OBJECTIVES:**

The escalating burden of cardiovascular disease (CVD) is a critical public health issue worldwide. CVD, especially acute myocardial infarction (AMI) and stroke, is the leading contributor to morbidity and mortality in Korea. We aimed to develop algorithms for identifying AMI and stroke events from the National Health Insurance Service (NHIS) database and validate these algorithms through medical record review.

**METHODS:**

We first established a concept and definition of “hospitalization episode,” taking into account the unique features of health claims-based NHIS database. We then developed first and recurrent event identification algorithms, separately for AMI and stroke, to determine whether each hospitalization episode represents a true incident case of AMI or stroke. Finally, we assessed our algorithms’ accuracy by calculating their positive predictive values (PPVs) based on medical records of algorithm-identified events.

**RESULTS:**

We developed identification algorithms for both AMI and stroke. To validate them, we conducted retrospective review of medical records for 3,140 algorithm-identified events (1,399 AMI and 1,741 stroke events) across 24 hospitals throughout Korea. The overall PPVs for the first and recurrent AMI events were around 92% and 78%, respectively, while those for the first and recurrent stroke events were around 88% and 81%, respectively.

**CONCLUSIONS:**

We successfully developed algorithms for identifying AMI and stroke events. The algorithms demonstrated high accuracy, with PPVs of approximately 90% for first events and 80% for recurrent events. These findings indicate that our algorithms hold promise as an instrumental tool for the consistent and reliable production of national CVD statistics in Korea.

## GRAPHICAL ABSTRACT

[Fig f2-epih-46-e2024001]

## Key Message

In this study, we developed algorithms to identify acute myocardial infarction (AMI) and stroke events from the Korean National Health insurance Service database. To validate them, we conducted retrospective review of medical records across 24 hospitals throughout Korea. The overall positive predictive values for the first and recurrent AMI events were around 92% and 78%, respectively, while those for the first and recurrent stroke events were around 88% and 81%, respectively.

## INTRODUCTION

The escalating burden of cardiovascular disease (CVD) represents a critical public health issue worldwide. In Korea, CVD has been identified as the primary contributor to morbidity and mortality [1]. Notably, acute myocardial infarction (AMI) and stroke represent a substantial proportion of CVD-related fatalities and disabilities. Therefore, ongoing surveillance and systematic management of these conditions are imperative, which requires accurate quantification of their incidence within the Korean population.

Previous attempts to estimate the national incidence of CVD in Korea have encountered several constraints. Predominantly, these attempts were not serial analyses but one-time studies, providing only snapshots of the situation instead of continuous monitoring of the diseases. Moreover, these studies faced methodological limitations, including the inability to appropriately discern recurrent CVD events [2,3]. In light of these challenges, the Korean Disease Control and Prevention Agency has inaugurated the National Cardiovascular Disease Statistics Production System, which aims to reliably monitor the national incidence of CVD and its trends.

In Korea, the absence of dedicated registries or cohorts with sufficient representativeness poses a significant challenge to the accurate estimation of the national incidence of CVD, which constitutes a significant gap in the country’s epidemiological resources. A potential alternative could be the National Health Insurance Service (NHIS) database, which encompasses the entire Korean population [4]. However, the NHIS database is primarily structured for administrative purposes and not explicitly designed for research applications. As such, the accurate estimation of CVD incidence requires the employment of specialized methodologies tailored to the unique structure and characteristics inherent to health claims databases.

Addressing this requirement, we have developed specific algorithms designed to identify both first-time and recurrent occurrences of AMI or stroke events based on the NHIS database. Our intention was to generate a more precise estimation of CVD incidence within the national context. To ensure the validity of our methodology, we retrospectively reviewed medical records from 24 hospitals across Korea. This extensive validation process is an essential step toward ensuring that our algorithm-driven approach can reliably capture the true incidence of CVD in the country.

## HOSPITALIZATION EPISODE

In health insurance claims data, it is not unusual to find instances where multiple claims are filed for a single disease episode [5]. This can occur in various circumstances, such as during extended hospital stays or in cases involving recurrent hospital admissions due to complications. For example, when a patient is hospitalized for AMI and the hospitalization exceeds a 30-day period, the insurance claims for the event are submitted in distinct 30-day intervals. Similarly, if a patient experiences stroke, is discharged, but is readmitted due to ensuing complications, multiple insurance claims are filed for what is essentially a single stroke event. Given that the claim codes for drug prescriptions, diagnostic tests, or medical procedures may be distributed across these separate claims, it becomes essential to consolidate all interrelated insurance claims into a single disease episode. This approach is particularly crucial when tracking recurrent events, as subsequent claims linked to the initial event can be misinterpreted as distinct events if not correctly merged with the original claim.

**Figure 1.**
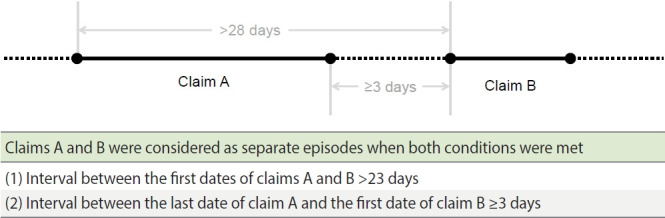
Definition of hospitalization episode.

As such, we have introduced the concept of a “hospitalization episode” to address these complexities. This term, as outlined in Figure 1, refers to the period covered by claims that can be reasonably attributed to a single disease event. Specifically, any 2 consecutive insurance claims A and B (both containing diagnosis codes for the disease of interest) were separated into distinct hospitalization episodes if: (1) there was a gap exceeding 28 days between the first dates of claims A and B, and (2) the interval between the last date of claim A and the first date of claim B spanned 3 days or longer. In other words, a sequence of insurance claims for a disease was defined as a single hospitalization episode if no consecutive pair in the sequence met the conditions for episode separation. This method allows for clear differentiation between distinct disease events and thus promotes a more accurate estimation of disease incidence and recurrence.

## ALGORITHMS FOR IDENTIFYING DISEASE EVENTS

Our identification algorithms for AMI and stroke events—developed over multiple internal meetings and confirmed after an external advisory meeting—primarily rely on International Classification of Diseases, 10th revision (ICD-10) diagnosis codes (AMI: I21-I23; stroke: I60, I61, I63, I64), supplemented by diagnostic test and/or procedure codes. Since the diagnosis codes are often carried over to subsequent hospital visits irrespective of the main purpose of the visit, the accuracy of the codes is generally much lower for identifying recurrent events than for first events. The algorithms were thus developed separately for the first and recurrent events, with the recurrent event identification algorithm incorporating more stringent additional criteria. We defined the first AMI or stroke event as the first hospitalization episode that met the criteria of our first event identification algorithm. Any subsequent hospitalization episodes that met our recurrent event identification algorithm’s criteria were recognized as recurrent events.

### First acute myocardial infarction identification algorithm

The accuracy of hospitalization diagnosis codes for AMI (ICD10; I21-I23) has been reported to be high when these codes appear in the primary position for the first time [6,7]. Therefore, in this case, any additional piece of evidence for AMI, such as the performance of electrocardiography, cardiac enzyme test, coronary angiography (CAG), percutaneous coronary intervention (PCI), or coronary artery bypass grafting (CABG), was deemed sufficient to classify the corresponding episode as the first AMI event. In addition, to account for situations in which patients die before any medical tests or procedures can be conducted (i.e., type 3 myocardial infarction), we also considered an in-episode death, defined as a death occurring during the episode, as additional evidence of AMI. In contrast, if the diagnosis codes were in the secondary or lower position, the performance of a diagnostic or therapeutic intervention (CAG, PCI, or CABG) that provides confirmatory evidence for AMI was deemed necessary to classify the corresponding episode as the first AMI event (Table 1).

### Recurrent acute myocardial infarction identification algorithm

Given the low accuracy of diagnosis codes in subsequent hospitalizations, the performance of a therapeutic intervention (PCI or CABG) that provides highly confirmatory evidence for AMI was regarded as necessary to classify the corresponding episode as a recurrent AMI event. Moreover, to reduce the possibility of incorrectly identifying stable or unstable angina events as recurrent AMI events, we incorporated an additional criterion that the episode length must be 3 days or longer unless interrupted by in-episode death (Table 1).

### First stroke identification algorithm

The accuracy of hospitalization diagnosis codes for stroke (ICD-10; I60-I61, I63-I64) has been reported to be fair, specifically when the codes appear in the primary position (for I63-I64) or in any position (for I60-I61) for the first time [2,6,7]. These codes, however, do not distinguish between acute and non-acute stroke. Accordingly, for the cases mentioned above, we considered the hospitalization episode as the first stroke event only if there was additional evidence suggesting a typical course of hospitalization for an acute stroke event. Such evidence included (1) the performance of brain imaging and an episode length of 3 days or longer unless interrupted by in-episode death, and (2) the performance of a therapeutic intervention. Moreover, considering situations where patients die before any brain imaging or therapeutic intervention, we treated in-episode death as additional evidence of stroke. In contrast, if the diagnosis codes I63-I64 were in the secondary position or lower, the performance of a therapeutic intervention and an episode length of 3 days or longer unless interrupted by in-episode death were required to classify the corresponding episode as the first stroke event (Table 2).

### Recurrent stroke identification algorithm

Considering the lower accuracy of diagnosis codes in subsequent hospitalizations, we used more stringent criteria in the recurrent stroke identification algorithm than in the first stroke identification algorithm. Regardless of the position of the diagnosis codes (I60-I61, I63-I64), an episode length of 3 days or longer or an in-episode death was required to classify the corresponding episode as the recurrent stroke event. If the diagnosis code appeared in the primary position, the performance of a brain imaging or therapeutic intervention was used as an additional criterion. If the diagnosis code appeared in the secondary position or lower, the performance of a therapeutic intervention was used as an additional criterion (Table 2).

## VALIDATION OF THE IDENTIFICATION ALGORITHMS

The AMI and stroke identification algorithms were validated through a retrospective review of medical records. Out of all the events identified by the respective algorithms, a total of 1,399 AMI events (median age at event, 68 years; 70.7% males) and 1,741 stroke events (median age at event, 71 years; 52.6% males) were sampled from 24 hospitals across Korea for medical record review (Table 3). The events were randomly selected after age-stratification and sex-stratification to ensure a representative sample of the total AMI and stroke events in Korea.

To verify whether each event identified by the algorithm indeed represented a true incident case of AMI or stroke, we established standardized epidemiological adjudication criteria for AMI and stroke. These criteria were developed through a series of internal meetings and finalized in an external advisory meeting, all before the commencement of the first medical record review.

### Epidemiological adjudication criteria for acute myocardial infarction

The epidemiological adjudication criteria for AMI were adapted from the 4th Universal Definition of Myocardial Infarction (UDMI) [8]. Briefly, the epidemiological adjudication criteria consisted of two major axes of acute myocardial injury and clinical ischemia conditions, slightly modified from those of the 4th UDMI considering the retrospective nature of the medical record review.

For adjudicating acute myocardial injury, we utilized not only cardiac troponins but also creatine kinase-myocardial band (CK-MB), given the widespread use of CK-MB and the potential preference for CK-MB over cardiac troponins in Korea. For adjudicating clinical ischemia, we modified the coronary thrombus criterion of the 4th UDMI to angiographic evidence of coronary artery disease considering the extremely low rate of autopsy in Korea. Moreover, we omitted the imaging evidence criterion—new loss of viable myocardium or new regional wall motion abnormality in a pattern consistent with an ischemic etiology—of the 4th UDMI to ensure that our AMI adjudication criteria would be equally applicable across all types of hospitals, including primary care hospitals where echocardiography is not widely implemented. The clinical ischemia condition was adjudicated based on the “highest” result among the remaining three criteria (angiographic evidence, ischemic symptoms, and electrocardiographic evidence).

The final epidemiological adjudication for AMI was performed by combining the acute myocardial injury condition and the clinical ischemia condition.

### Epidemiological adjudication criteria for stroke

The epidemiological adjudication criteria for stroke were adapted from the 2013 American Heart Association (AHA)/American Stroke Association (ASA) definition of stroke [9]. Briefly, the epidemiological adjudication criteria consisted of one major axis of objective evidence condition, slightly modified from that of the 2013 AHA/ASA definition considering the retrospective nature of the medical record review. The clinical evidence condition for ischemic stroke has been omitted given the potential subjectivity of neurological symptom/sign evaluation and reporting and the growing importance of objective evidence for stroke diagnosis. The objective evidence condition was adjudicated using the first 3 readings for each of brain computed tomography, brain magnetic resonance imaging, and cerebral angiography conducted during the hospitalization. The final epidemiological adjudication for stroke was performed according to the objective evidence condition.

### Retrospective medical record review and assessment of the positive predictive value

We conducted retrospective medical record reviews for a total of 3,140 algorithm-identified events (1,399 AMI and 1,741 stroke) at 24 hospitals (5 tertiary, 10 secondary, and 9 primary care hospitals) throughout the nation (Table 3). The positive predictive values (PPVs) were calculated as (the number of algorithm-identified events adjudicated as a true case)/(the number of algorithm-identified events examined)× 100%, separately for the first and recurrent events and types of hospitals (tertiary, secondary, and primary). We calculated both the unweighted pooled PPV, which involved simple aggregation, and the weighted pooled PPV, considering the number of cases reported per hospital type from 2011 to 2020. When calculating the weighted pooled PPV, for cases involving multiple hospital visits, the calculations were conducted separately, based on the first hospital visited during the episode period, and also based on the highest-level hospital visited.

The weighted PPVs based on first-visit and highest-level hospitals during first AMI event episode were 92.0% and 92.2%, respectively. Furthermore, the weighted PPVs based on first-visit and highest-level hospitals during recurrent AMI event episodes were 77.8% and 78.2%, respectively (Table 4). The weighted PPVs based on first-visit and highest-level hospitals during first stroke event episode were 88.2% and 88.1%, respectively. Furthermore, the weighted PPVs based on first-visit and highest-level hospitals during recurrent stroke event episodes were 80.8% and 81.0%, respectively (Table 5).

## DISCUSSION

As our effort to assess the national incidence of CVD, we developed and validated methods to identify incident AMI and stroke events using the NHIS database. These methods involved grouping individual hospitalization claims into hospitalization episodes and subsequently determining whether each episode met our AMI or stroke identification algorithms. The development process prioritized the sustainability and manageability of our algorithms, the qualities essential for a consistent replication of annual CVD statistics in Korea. We further validated these algorithms via a retrospective review of over 3,000 medical records from 24 hospitals nationwide.

The PPVs of our identification algorithms were comparable to those of other algorithms or definitions reported in previous studies [2,6,10,11]. Our algorithms are unique, however, in their ability to identify not only the first events but also recurrent events. Most previous attempts to identify recurrent CVD events have largely failed due to the substantial decline in diagnostic code accuracy upon subsequent hospital visits. In addition, the fact that a single disease event often invokes multiple insurance claims rendered such efforts more challenging. To address these issues, we introduced and defined the concept of “hospitalization episode,” whereby consecutive health insurance claims are grouped into a single disease episode under specific conditions. This approach enabled us to simplify our recurrent event algorithms while sustaining high PPVs.

Our AMI and stroke identification algorithms also have several limitations. First, the NHIS database does not include individuals who developed AMI or stroke but did not, or could not, utilize medical services. This is particularly relevant for recurrent AMI, as a considerable number of patients with stent thrombosis may die before arriving at the hospital [12,13]. Second, the NHIS database does not include cases where insurance claims were not filed for medical services that were actually utilized. This exclusion extends to drug prescriptions, examinations, or procedures that are not covered by the NHIS scheme [4]. Third, given the claims-based nature of the database, our algorithms are vulnerable to shifts in medical policies, reimbursement criteria, and variations in diagnosis coding and claiming patterns. For instance, the introduction of the 7th revision of the Korean Standard Classification of Diseases in 2016 may have affected the apparent sudden changes in the incidence of AMI and stroke that year. The diagnoses and claims patterns may also have been impacted by the coronavirus disease 2019 pandemic in 2020.

## CONCLUSION

We have established a concept of “hospitalization episode” and developed algorithms to identify both first and recurrent events of AMI or stroke. The goal was to estimate Korea’s national CVD incidence utilizing the NHIS database. Despite some limitations, primarily associated with the claims-based nature of the database, these algorithms achieved PPVs of approximately 90% for first events and 80% for recurrent events. They promise to be instrumental tools in reliably and consistently generating national CVD statistics in Korea.

### Ethics statement

This study complied with the Declaration of Helsinki, and the study protocol was approved by the Institutional Review Board (IRB) of Yonsei University Health System, Seoul, Korea (#4-2022-0586). Informed consent was waived by the IRB. Informed consent was waived since this is a retrospective study of de-identified administrative data.

## Figures and Tables

**Figure f2-epih-46-e2024001:**
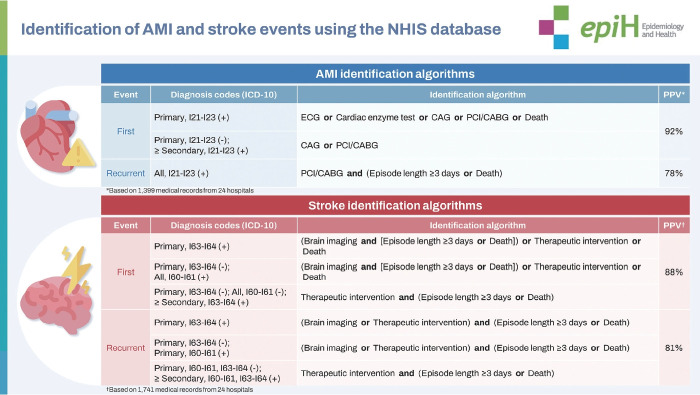


**Table 1.** Identification algorithms for first and recurrent acute myocardial infarction events

Event	Diagnosis codes (ICD-10)	Identification algorithm
First	Primary, I21-I23 (+)	ECG or cardiac enzyme test or CAG or PCI/CABG or death
	Primary, I21-I23 (-); Secondary or lower, I21-I23 (+)	CAG or PCI/CABG
Recurrent	All, I21-I23 (+)	PCI/CABG and (episode length ≥3 days or death)

ICD-10, International Classification of Diseases, 10th revision; ECG, electrocardiogram; CAG, coronary angiography; PCI, percutaneous coronary intervention; CABG, coronary artery bypass grafting.

**Table 2.** Identification algorithms for first and recurrent stroke events

Event	Diagnosis codes (ICD-10)	Identification algorithm
First	Primary, I63-I64 (+)	(Brain imaging and [episode length ≥3 days or death]) or therapeutic intervention^1^ or death
	Primary, I63-I64 (-); All, I60-I61 (+)	(Brain imaging and [episode length ≥3 days or death]) or therapeutic intervention^1^ or death
	Primary, I63-I64 (-); All, I60-I61 (-); Secondary or lower, I63-I64 (+)	Therapeutic intervention^1^ and (episode length ≥3 days or death)
Recurrent	Primary, I63-I64 (+)	(Brain imaging or therapeutic intervention^1^) and (episode length ≥3 days or death)
	Primary, I63-I64 (-); Primary, I60-I61 (+)	(Brain imaging or therapeutic intervention^1^) and (episode length ≥3 days or death)
	Primary, I60-I61, I63-I64 (-); Secondary or lower, I60-I61, I63-I64 (+)	Therapeutic intervention^1^ and (episode length ≥3 days or death)

ICD-10, International Classification of Diseases, 10th revision.

1 Including intravenous thrombolysis, endovascular treatment, coil embolization, or other specific therapeutic interventions for stroke.

**Table 3.** Number of medical record review by hospital type

Hospital type	Center	No. of cases reviewed	
		AMI	Stroke
Tertiary	A	421	428
	B	20	20
	C	108	103
	D	86	89
	E	90	90
	Subtotal	725	730
Secondary	F	90	99
	G	40	36
	H	39	41
	I	41	33
	J	40	40
	K	120	118
	L	30	28
	M	49	45
	N	70	59
	O	33	30
	Subtotal	552	529
Primary	P	3	17
	Q	17	140
	R	23	36
	S	31	18
	T	12	168
	U	17	22
	V	7	32
	W	2	21
	X	10	28
	Subtotal	122	482
Total		1,399	1,741

AMI, acute myocardial infarction.

**Table 4.** PPV of AMI identification algorithms

Hospital type	First AMI event			Recurrent AMI event		
	Adjudicated	Identified	PPV (95% CI), %	Adjudicated	Identified	PPV (95% CI), %
Tertiary	584	617	94.7 (92.9, 96.4)	88	108	81.5 (74.2, 88.8)
Secondary	339	368	92.1 (89.4, 94.9)	138	184	75.0 (68.7, 81.3)
Primary	51	121	42.1 (33.4, 50.9)	0	1	N/A
Weighted 1^1^	-	-	92.0 (89.5, 94.4)	-	-	77.8 (71.0, 84.7)
Weighted 2^2^	-	-	92.2 (89.9, 94.6)	-	-	78.2 (71.4, 85.0)

PPV, positive predictive value; AMI, acute myocardial infarction; CI, confidence interval; N/A, not available.

1 Based on first-visit hospital within an episode.

2 Based on highest-level hospital within an episode.

**Table 5.** PPV of stroke identification algorithms

Hospital type	First stroke event			Recurrent stroke event		
	Adjudicated	Identified	PPV (95% CI), %	Adjudicated	Identified	PPV (95% CI), %
Tertiary	458	549	83.4 (80.3, 86.5)	137	181	75.7 (69.4, 81.9)
Secondary	335	356	94.1 (91.7, 96.5)	154	173	89.0 (84.4, 93.7)
Primary	275	392	70.2 (65.6, 74.7)	57	90	63.3 (53.4, 73.3)
Weighted 1^1^	-	-	88.2 (85.1, 91.3)	-	-	80.8 (74.8, 86.9)
Weighted 2^2^	-	-	88.1 (85.0, 91.1)	-	-	81.0 (75.0, 87.0)

PPV, positive predictive value; CI, confidence interval.

1 Based on first-visit hospital within an episode.

2 Based on highest-level hospital within an episode.
